# Clinical and radiographic analysis of unilateral versus bilateral instrumented one-level lateral lumbar interbody fusion

**DOI:** 10.1038/s41598-020-59706-9

**Published:** 2020-02-20

**Authors:** Masayoshi Fukushima, Yasushi Oshima, Yohei Yuzawa, Sakae Tanaka, Hirohiko Inanami

**Affiliations:** 1 Inanami Spine and Joint Hospital, Tokyo, Japan; 2 Department of Orthopaedic Surgery, The University of Tokyo, Tokyo, Japan

**Keywords:** Spinal cord diseases, Outcomes research

## Abstract

Lateral lumbar interbody fusion (LLIF) is a widely applied and useful procedure for spinal surgeries. However, posterior fixation has not yet been decided. We compared the radiographic and clinical outcomes of unilateral versus bilateral instrumented one-level LLIF for degenerative lumbar disease. We conducted a prospective cohort study of 100 patients, who underwent unilateral (group U) or bilateral (group B) instrumented one-level LLIF for degenerative lumbar disease. Forty-one patients in group U were undergoing unilateral pedicle screw instrumentation, and 59 patients in group B were undergoing bilateral pedicle screw instrumentation. Clinical characteristic and demographic data before surgery were compared. The intraoperative data, including operative time with changes in positions, intraoperative blood loss, and X-ray exposure time, as well as the perioperative data, including postoperative hospital stay and clinical and radiographic data were compared. As a result, Group U required a significantly shorter operating time than group B. The subsidence grade and fusion rates exhibited no significant differences in the postoperative radiographic evaluation. Group U had better results in clinical assessments than group B. However, group U required more additional surgeries owing to complications.

## Introduction

A number of techniques have been developed for lumbar spinal fusion. Recently, interbody fusion has become widely popular as it provides a large surface area for fusion with the graft between adjacent vertebral bodies^1^. In the past, the approach to the anterior column of the lumbar spine for interbody fusion was limited to an anterior or posterior procedure^2–4^. Ozgur *et al*. introduced transpsoas lateral lumbar interbody fusion (LLIF) as a minimally-invasive alternative to the traditional anterior and posterior approach^5^. Due to its minimally-invasive nature, LLIF has become a popular procedure for spinal surgeries today, and it is applied to a wide range of spinal conditions^6–8^.

In comparison with conventional lumbar interbody fusion, LLIF has some advantages including large discectomy, bilateral annular release, preservation of both anterior and posterior longitudinal ligaments, preservation of posterior bony structures and insertion of large grafts. For these reasons, LLIF is thought to be inherently more stable than alternative stand-alone anterior fusion. Therefore, there are a few clinical studies that have evaluated the outcomes of patient underwent stand-alone LLIF without pedicle screw insertion^7,9^.

The posterior lumbar interbody fusion (PLIF) or transforaminal lumbar interbody fusion (TLIF) were performed typically in conjunction with pedicle screw due to the limitation of graft size^10,11^. This prompted discussion on the methods of posterior fixation in relation to PLIF or TLIF^12–14^. Many studies have compared the effectiveness of TLIF with unilateral and bilateral instrumentation^15–18^. On the other hand, the need for posterior fixation in LLIF procedure has not yet been decided. The procedure of LLIF performed in the lateral decubitus position inevitably requires a posture change to insert the bilateral percutaneous pedicle screw (PPS). Therefore, if LLIF with unilateral instrumentation can obtain similar results to bilateral instrumentation, this would greatly simplify the procedure and obviate a posture change. Currently, there was a few studies evaluating clinical outcomes about LLIF with unilateral instrumentation^19,20^. However, there have been no reports which directly compared clinical outcomes of LLIF between unilateral and bilateral instrumentation. Therefore, the purpose of this study was to compare the radiographic and clinical outcomes of unilateral versus bilateral instrumented one-level LLIF for degenerative lumbar disease.

## Material and Methods

This study was approved by the ethical committee of Iwai Medical Foundation at authors’ institution, and written informed consent was obtained from all participants. As agreed with the ethical committee, all methods were performed in accordance with the relevant guidelines and regulations. We conducted a retrospective review of inpatient medical records, preoperative and postoperative outpatient clinical charts at our hospital. Records of 128 patients, covering the period from May 2013 to April 2015, were reviewed. All patients had suffered from low back pain, severe radicular pain, or neurologic symptoms. Patients who had undergone surgical treatments had also undergone conservative treatments prior to surgery. We decided to perform the LLIF procedure with unilateral PPS for the first consecutive 50 cases and then change to the procedure with bilateral PPS for the subsequent 78 cases. In total, 41 of the first consecutive 50 patients who underwent LLIF with unilateral PPS (group U) for degenerative lumbar disease over the 12-month period from May 2013 fulfilled the below inclusion criteria. These 41 patients were compared with the subsequent consecutive 59 patients who underwent LLIF with bilateral PPS (group B) from April 2014 and fulfilled with the inclusion criteria in a historical comparative analysis. This study only included patients with one-level degenerative disease. Inclusion criteria were: one-level lumbar spinal stenosis with spondylolisthesis (more than Meyerding grade I) or lumbar instability including restenosis after decompression. Lumbar instability was defined as more than 3 mm of translation or 15 degrees of angular motion on preoperative flexion-extension radiographs^21^. Exclusion criteria were: history of other spinal surgical treatments, vertebral body fracture proximal to the location of interbody fusion, neuromuscular disease, scoliosis (Cobb angle ≥10° on neutral radiographs) and hyper degeneration which required at least two-level interbody fusion.

The data collected for analysis were operative time (including time for surgical repositioning if needed), intraoperative blood loss, X-ray exposure time, duration of postoperative hospital stay, postoperative radiographic results, and complications.

All surgical procedures were performed in accordance with ordinary LLIF procedure guidelines previously reported^5^. In this study, we used PEEK cage filled with collagen hybrid hydroxyapatite in all the patients. We performed all the LLIF procedures in all the patients using the fluoroscopy. In regards to posterior fixation, we inserted the cage into the interbody in the lateral decubitus position. In the U group, we performed the insertion of PPS to the ipsilateral side of the cage insertion while remaining in the lateral decubitus position. Conversely, in group B we performed insertion of bilateral PPS after surgical repositioning from lateral to prone following cage procedure to insert bilateral PPS correctly using fluoroscopy. Therefore, we included time for surgical repositioning in the operative time. Postoperatively, soft bracing was used for at least three months in all the patients.

### Radiographic assessment

**Figure 1:**
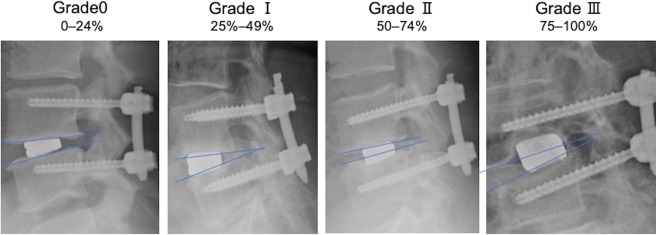
Subsidence grading. Radiographs showing examples of the different subsidence grades (0-III).

Serial radiographs (neutral and dynamic standing films), CT scans, and magnetic resonance imaging were analyzed for the purposes described below. One spine surgeon with over clinical experience evaluated radiographic assessment. Intervertebral disc height was measured using standing neutral lateral radiographs, evaluated as an average of the anterior and posterior margins of the intervertebral space. Segmental lordosis was determined by the angle between lines perpendicular to the inferior endplate of the superior vertebra and the superior endplate of the inferior vertebra in each treated level. Segmental lordosis was determined both on forward and backward bending to assess instability of the intervertebral disc. Subsidence was measured from standing neutral lateral radiographs with parallel endplates at the index level. The degree of vertebral body collapse around the disc space was then categorized (Fig. 1): Grade 0 = 0–24%, Grade I = 25–49% collapse, Grade II = 50–74% collapse, and Grade III = 75–100% collapse^9^. Subsidence was evaluated at the 12-month visit or during the re-operation period, if needed. Fusion was evaluated 12-months postoperatively, and was defined as bridging bone connecting the adjacent vertebral bodies either through or around the implants, <5° of angular motion, ≤3 mm of translation on the radiography, and absence of radiolucent lines >50% of either of the implant surfaces on CT scans^22^.

### Clinical assessment

Clinical assessments were performed preoperatively and at every routine postoperative follow-up (3 weeks, 3 months, 6 months and 12 months) in the outpatient clinic. The Zurich claudication questionnaire (ZCQ), which consists of symptom severity and functional disability, and the Japanese Orthopaedic Association score (JOA Score) was used preoperatively and at the 12-month visit for the patients whom we could observe. We evaluated the patients who required additional surgery at the 12-month after first surgery. Postoperative complications were recorded at each follow-up.

### Statistical analysis

We compared the differences in these parameters between the two groups. Statistical evaluation was performed using JMP software version 12.0 (SAS Institute Inc., Cary, North Carolina, USA). The Wilcoxon rank-sum test was used for the analysis of quantitative data in the evaluation of demographic and outcome measures between the two groups. A comparative study of the ratio of the patients with fusion was done by a Fisher’s exact test. A *p* value less than 0.05 was considered statistically significant.

## Results

The demographic data for each group are listed in Table 1. In the U group, 41 patients underwent unilateral PPS instrumentation, and in group B, 59 patients underwent bilateral PPS instrumentation. There was no difference in mean age between groups (*p* = 0.32). Sex (*p* = 0.69), BMI (*p* = 0.71) and operated segments (0.87) were not different between groups.

**Table 1:** Demographic data of two groups.

	Group U (n = 41)	Group B (n = 59)	*p*
Mean age (range)	60.1 (21–84)	63.2 (39–90)	0.32
Sex (M/F)	19 / 22	25 / 34	0.69
Mean height (cm) (range)	163.8 (146.5–180.7)	165.0 (136.2–182.1)	0.43
Mean mass (Kg) (range)	65.2 (39.8–102.3)	67.2 (39.3–94.6)	0.63
Mean BMI (Kg/m^2^) (range)	23.9 (17.0–33.4)	24.0 (18.1–38.2)	0.71
Operated segments			0.87
L3-L4	9	14	
L4-L5	32	45	

Group U required significantly shorter operating time than group B (*p* = 0.003). There was no difference in amount of blood loss, X-ray exposure time, or postoperative hospital stay between the two groups in first surgery. (Table 2)

**Table 2:** Comparison of clinical data between two groups.

	Group U (n = 41)	Group B(n = 59)	*p*
Mean Operative time (min) (range)	83.1 (42–216)	119.2 (60–277)	0.003
Mean Intraoperative blood loss (ml) (range)	59.1 (7–110)	52.1 (10–140)	0.35
Mean X-ray exposure time (sec) (range)	288.7 (168–609)	293.9 (19–929)	0.80
Mean Duration of postoperative hospital stay (day) (range)	14.7 (9–26)	15.1 (5–27)	0.16

The radiographic outcomes were listed in Table 3. There were no significant differences in relation to subsidence at 12 months after surgery (*p* = 0.72). Fusion rate was judged on the radiographs 12 months postoperatively. A total of 36 cases in group U and 50 cases in group B achieved fusion at 12 months post-surgery. The fusion rate was 87.8% in group U and 84.7% in group B with no statistical difference (*p* = 0.77).

**Table 3:** Comparison of radiological complications between the two groups.

	Group U (n = 41)	Group B (n = 59)	*p*
Subsidence			0.72
Grade 0	22	30	
Grade I	12	19	
Grade II	7	10	
Grade III	0	0	
Fusion rate (%)	36 (87.8%)	50 (84.7%)	0.77
Additional surgery	5	1	0.018

The mean value of ZCQ and JOA scores between pre- and post- operative time in both groups are shown in Table 4. Each postoperative score significantly improved after surgery in both groups (*p* < 0.01). Comparison of clinical assessments between the two groups in pre- and post- operative time are shown in Table 5. Group U had better results in ZCQ symptom severity and JOA score at 12 months after surgery than group B.

**Table 4:** Comparison of clinical assessments between between pre- and post- operative scores in both groups.

	Pre OP	12-month post OP	Treatment Effect (95% Cl)	*p*
**ZCQ symptom severity**				
Group U	3.09	2.13	−0.97 (−0.67 to −1.29)	<0.001
Group B	3.17	2.66	−0.49 (−0.22 to −0.89)	<0.001
**ZCQ functional disability**				
Group U	2.22	1.58	−0.67 (−0.35 to −0.94)	<0.001
Group B	2.38	2.01	−0.46 (−0.29 to −0.9)	<0.001
**JOA score**				
Group U	10.85	16.14	5.2 (3.72 to 7.61)	<0.001
Group B	10.46	12.91	2.45 (1.44 to 6.20)	<0.001

ZCQ Zurich claudication questionnaire, JOA Score Japanese Orthopaedic Association score.

**Table 5:** Comparison of clinical assessments between the two groups.

	Group U	Group B	
**ZCQ symptom severity**			
Pre op	3.09	3.17	0.50
12-month post OP	2.13	2.66	0.028
**ZCQ functional disability**			
Pre op	2.22	2.38	0.46
12-month post OP	1.58	2.01	0.019
**JOA Score**			
Pre op	10.85	10.46	0.73
12-month post OP	16.14	12.91	0.029

ZCQ Zurich claudication questionnaire, JOA Score Japanese Orthopaedic Association score.

Six (6.0%) patients required additional surgery on average 5.2 months after surgery (range: 2–10 months). Additionally, significant differences were observed in the requirement for additional surgery. In group U, five patients required additional surgeries because of loosening of the cage, vertebral body fracture, or infection, whereas in group B, one patient required additional surgery because of adjacent segmental disease with herniation. We provide details about these additional surgeries in Table 6. There were three patients in group U who felt worsening of low back pain revealed loosening by radiographic examination. Therefore, we diagnosed their low back pain as caused by loosening. One patient felt low back pain caused by caudal vertebral body fracture in the fused intervertebral body at 4-months after surgery. And, another patient developed a fever at 2-months after surgery along with low back pain. On the other hand, there was one patient in group B who felt new neurological symptoms after surgery caused by adjacent segmental disease with herniation. The patient was finally treated with new interbody fusion for the segment approached by the TLIF procedure with extended bilateral PPS.

**Table 6:** The details about the additional surgeries after one-level LLIF.

Case No.	Sex	Age	Cause of the revision surgery	Duration since the first surgery	Method for the revision surgery
**Group U**					
1	F	84	loosening of the cage	4 Months	contralateral PPS
2	F	65	loosening of the cage	6 Months	contralateral PPS
3	M	78	caudal vertebral body fracture	4 Months	contralateral PPS
4	M	35	loosening of the cage	5 Months	contralateral PPS
5	M	74	infection	2 Months	Irrigation and contralateral PPS
**Group B**					
1	F	72	adjacent segmental disease with herniation	10 Months	TLIF procedure with extended bilateral PPS

### Illustrative cases

**Figure 2:**
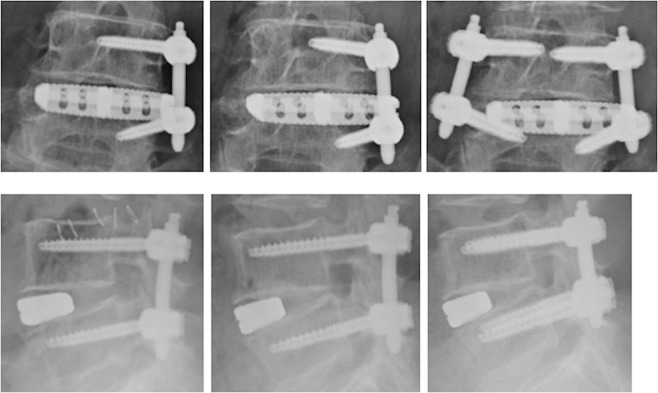
Radiographs of a 72-year-old woman showing after surgery at L4–5; LLIF with unilateral PPS. After 3 months, radiographs revealed loosening of the cage. After 18 months from re-operation, radiographs showed positions remained unchanged.

A 72-year-old woman with neurogenic claudication failed conservative treatment. Magnetic resonance imaging revealed a grade I spondylolisthesis of L4 which caused severe canal stenosis at same level with instability on dynamic radiography. Surgical treatment included LLIF at L4/5, followed by single-level unilateral PPS. Postoperative imaging confirmed disc height restoration, realignment and screw positioning in the correct place. (Fig. 2) Three months after the surgery, she reported low back pain, which she had never suffered from, without neurological symptoms. Radiographs revealed loosening of the cage at the lateral insertion with interbody graft subsidence. Her symptoms persisted despite conservative treatment, including bed rest with brace. Therefore, revision surgery was performed by adding a contralateral PPS without revising the LLIF cage. At 18-months postoperative, she could move without any symptoms and the radiographic exams revealed an unchanged LLIF cage and PPS.

## Discussion

We sought to clarify clinical and radiographic outcomes of LLIF with unilateral versus bilateral PPS and revealed two main findings. First, the additional surgery rate was higher in patients underwent unilateral PPS, although the overall fusion rate was similar to bilateral PPS. Second, LLIF with unilateral PPS required shorter operating time and showed better clinical outcomes.

In spinal fusion surgery, the need for unilateral or bilateral pedicle screw instrumentation for lumbar interbody fusion has been a controversial issue. In the past, many clinical studies have compared the need for unilateral or bilateral pedicle screw instrumentation for the posterior approach lumbar interbody fusion^15,16^. At present, the general consensus is that unilateral pedicle screw instrumentation should be confined to a one-level posterior approach lumbar interbody fusion and not be extended to multilevel fusion because of inadequate fixation strength^23–26^.

However, there have been no clinical studies comparing the differences between the unilateral or bilateral PPS for LLIF procedure. On the other hand, there are several biomechanical studies using cadaveric spine models which compared the stability of the LLIF with or without posterior instrumentation. These studies showed that both the addition of unilateral and bilateral PPS produced a significant decrease in range of motion, compared with the standalone cages, in all axes of motion^27–30^. Finally, they concluded that surgeons should choose fixation options commensurate with the stability requirements of individual patients. In our study, in relation to the radiographic assessment there were no significant clinical differences between the two groups regarding the subsidence and fusion status at 12-months postoperatively. Therefore, we suggest that unilateral PPS should be confined to a one-level LLIF from this study’s outcomes.

However, this study showed that LLIF with unilateral PPS required additional surgery owing to the greater rate of complications. The need for additional surgery in the bilateral PPS resulted from adjacent segmental disease. Conversely, additional surgery in the unilateral PPS resulted from exacerbation at the surgical site. The three cases of additional surgery in the unilateral PPS (except one case of infection) were attributed to loosening of the cage, and the other case was due to caudal vertebral body fracture in the fused intervertebral body. In these four cases, inserting PPS in the contralateral side to reduce exacerbation at the surgical site could improve stability in radiographic assessment. These findings indicate that the LLIF with unilateral PPS did not have sufficient stability for the lumbar spine to be fused, compared with bilateral PPS. It is possible that we could not place sufficient compression force in the lateral position, as the lumbar alignment in lateral position would inevitably differ from the prone position. Furthermore, a past retrospective study suggested that the rate of graft failure following LLIF is inversely related to bone mineral density. In this study, three patients who required additional surgeries were older or female^31^. We could not measure bone mineral density in all the patients, but there was a possibility that bone mineral density contributed to the loosening of the cage.

This study identified that LLIF with unilateral PPS requires a significantly shorter operating time than bilateral PPS, as there is no need for posture change during LLIF with unilateral PPS. We performed all the LLIF procedures in all the patients in this study using the fluoroscopy in our facility. Therefore, we decided the repositioning from lateral to prone following insertion of the cage. If we could perform insertion of bilateral PPS as in navigation system while remaining in the lateral decubitus position, we may indicate that there is no significantly differences in both groups.

In addition, patients who underwent LLIF with unilateral PPS had better results at 12-months after surgery than patients with bilateral PPS. A prospective randomized study reported that unilateral instrumented TLIF was an effective and safe method with the advantages of reduced operative time and blood loss, avoiding damage to contralateral paravertebral muscles and bone structures, and minimal compromise of spinal stability^12^. We suggest that damage can be avoided by posterior fixation using unilateral PPS for LLIF procedure, and this may lead to better clinical results than bilateral PPS.

We showed that LLIF with unilateral PPS required a shorter operating time and showed better clinical outcomes than LLIF with bilateral PPS. We can choose unilateral instrumentation in the one-level LLIF procedure especially for patients with stronger bone mineral density. However, we could not determine why some patients experienced loosening of the cage in LLIF with unilateral PPS. Therefore, we shall continue to perform the LLIF procedure with bilateral PPS until the reason is determined.

There are several limitations to this study. First, LLIF with unilateral PPS was performed at an earlier stage of disease than bilateral PPS which might have influenced these results. Second, radiological assessments were performed by only one reader and had inherent errors. Third, we could not evaluate the preoperative dynamic factors for the interbody to determine the correct LLIF procedure based on bone mineral density condition. Morphological measurements may have been affected by the presence or absence of dynamic instability at the surgical intervertebral disc site. Finally, we could not evaluate the long-term outcomes for this research. Further studies will be necessary to elucidate these problems.

## Conclusion

LLIF with unilateral instrumentation required a significantly shorter operating time compared to bilateral instrumentation. Although the overall fusion rates were not significantly different, LLIF with unilateral instrumentation required more additional surgeries.
